# Efficacy and safety of tirabrutinib monotherapy in relapsed or refractory B-cell lymphomas/leukemia: a meta-analysis

**DOI:** 10.3389/fphar.2025.1559056

**Published:** 2025-04-01

**Authors:** Jingfei Wang, Hai-en Cheng, Yanhua Sun, Zhenfeng Li, Yunke Zang, Yuying Zhao, Guimao Yang, Zhijun Liu, Yaqi Sun, Yanli Sun

**Affiliations:** ^1^ School of Medical Laboratory, Shandong Second Medical University, Weifang, China; ^2^ Department of Hematology, Weifang People’s Hospital, Weifang, China; ^3^ Medicine Research Center of Shandong Second Medical University, Shandong Second Medical University, Weifang, China; ^4^ Department of Clinical Laboratory, Affiliated Hospital of Shandong Second Medical University, Weifang, China; ^5^ School of Basic Medicine, Shandong Second Medical University, Weifang, China

**Keywords:** Btk inhibitor, tirabrutinib, B-cell lymphomas/leukemia, safety, efficacy

## Abstract

**Background:**

Tirabrutinib is a potent, irreversible, and highly selective Bruton’s tyrosine kinase (BTK) inhibitor that has demonstrated significant therapeutic activity in patients with relapsed or refractory B-cell lymphoma. However, the safety and efficacy of tirabrutinib monotherapy have not been systematically assessed. This meta-analysis aims to assess the safety and efficacy of tirabrutinib monotherapy in patients with B-cell lymphoma.

**Methods:**

We conducted a comprehensive literature search in Embase, PubMed, Web of Science, and Cochrane Library databases to identify prospective clinical trials evaluating tirabrutinib monotherapy in B-cell lymphoma. Data on efficacy and safety were extracted and analyzed.

**Results:**

A total of seven studies were included in the meta-analysis. The clinical trials primarily involved patients with chronic lymphocytic leukemia (CLL), primary central nervous system lymphoma (PCNSL), mantle cell lymphoma (MCL), and Waldenström’s macroglobulinemia (WM). The pooled overall response rate (ORR) was 72.5%, with complete response (CR) rate of 18.6%, stable disease (SD) rate of 13.8%, and partial response (PR) rate of 41.1%. The highest median progression-free survival (mPFS) was observed in patients with CLL, reaching 38.5 months. Neutropenia was the most common adverse event (AE), both all grades and grade ≥3. Additionally, a high incidence of skin-related AEs was reported.

**Conclusion:**

Tirabrutinib monotherapy demonstrates a manageable safety profile and promising efficacy in patients with B-cell lymphoma. However, these findings need to be confirmed by larger and higher-quality randomized controlled trials in the future. Further research should also explore the long-term effects and potential benefits of combination therapies involving tirabrutinib.

**Systematic Review Registration:**

Identifier CRD42024552721.

## 1 Introduction

Lymphoma is a heterogeneous group of lymphoproliferative disorders (Liu et al., 2021) and is the most common hematologic malignancy worldwide (Yurkovski et al., 2023). In 2024, it is estimated that there will be 89,190 new cases and 21,050 deaths from lymphoma in the United States alone (Siegel et al., 2024). Mature B-cell tumors and T-cell tumors are the most typical forms of lymphoid malignancies following stem cell differentiation (Wang et al., 2023). Despite significant advances in supportive care and treatment strategies, overall survival rates have improved over the past decades (Levy et al., 2023), but unmet treatment needs remain, particularly for patients with Non-Hodgkin’s lymphoma (NHL). A large proportion of patients experience relapse or refractory disease after initial treatment (Klein et al., 2021).

Bruton’s tyrosine kinase, a non-receptor tyrosine kinase of the TEC family, plays a pivotal role in modulating the B-cell antigen receptor (BCR) signaling pathway, which is crucial for B-cell proliferation and survival. Targeting the BCR pathway has emerged as a promising therapeutic strategy for B-cell lymphomas, with BTK inhibitors at the forefront of this approach (Wang et al., 2021). The first-generation BTK inhibitor, ibrutinib, has demonstrated significant clinical activity in B-cell malignancies driven by active BCR signaling (Tanaka et al., 2020). However, its use is limited by off-target effects, which can lead to increased risks of bleeding, hypertension, and atrial fibrillation (Brown et al., 2023). This highlights the need for more effective and selective BTK inhibitors. Tirabrutinib, a second-generation BTK inhibitor, is a potent, irreversible, and highly selective inhibitor of BTK, which is essential for the proliferation and viability of cancerous B cells (Roufarshbaf et al., 2022). It has shown significant efficacy, particularly in patients with relapsed or refractory B-cell lymphoma (Liclican et al., 2020). This meta-analysis aims to provide a comprehensive evaluation of the safety and efficacy of tirabrutinib monotherapy in patients with B-cell lymphoma, offering evidence-based insights to guide future clinical trials and therapeutic protocols.

## 2 Methods

### 2.1 Literature search

The research and literature search strategies employed in this study adhered to the Preferred Reporting Items for Systematic Reviews and Meta-Analyses (PRISMA) guidelines. We conducted a comprehensive literature search in Embase, PubMed, Web of Science, and the Cochrane Library databases using the search terms “Tirabrutinib,” “GS-4059,” and “ONO-4059,” without applying any automated filters (e.g., date, language). The search was completed on 20 February 2024, and identified clinical trials that met the inclusion criteria and evaluated the efficacy and safety of tirabrutinib in the treatment of B-cell lymphoma.

### 2.2 Inclusion and exclusion criteria

The inclusion criteria for the trials were as follows: (1) clinical trials evaluating tirabrutinib for B-cell lymphoma at any stage; (2) tirabrutinib administered as monotherapy, irrespective of prior treatments; (3) availability of evaluable efficacy or safety data; and (4) relevance to human clinical trials.

The exclusion criteria for the trials were as follows: (1) clinical trials involving tirabrutinib in combination with other drugs; (2) trials that did not report any results; (3) article types such as reviews, commentaries, letters, and case reports; and (4) studies conducted in cells or animals.

Two authors independently conducted the literature search and screening, collaboratively assessed whether studies met the inclusion criteria, and resolved any discrepancies through discussion.

### 2.3 Data extraction

Two authors independently extracted data from the eligible studies, focusing on the following key data points: 1) Basic Information: Details of the included studies, including the first author, publication date, clinical trial registration number, phase of the clinical trial, study design, total number of patients enrolled, gender distribution, cancer type, and patient age; 2) AEs: Incidence of all grades AEs and grade ≥3 AEs; 3) Survival Endpoints: ORR, SD, CR, PR, and mPFS; and 4) Pharmacokinetic Parameters: Time to peak concentration (T_max_), terminal elimination half-life (T_1/2_), and maximum concentration (C_max_).

To comprehensively elucidate the targets of tirabrutinib and their biological roles, we conducted a detailed bioinformatics analysis. The target genes of tirabrutinib were identified through a thorough review of relevant research articles published in PubMed. In addition, the Kyoto Encyclopedia of Genes and Genomes (KEGG) pathway and Gene Ontology (GO) enrichment analysis were conducted using the bioinformatics website (http://www.bioinformatics.com.cn/). Additionally, to further elucidate the interactions among the target proteins of tirabrutinib, protein-protein interaction (PPI) networks were made using STRING (https://cn.string-db.org/). The networks were visualized and analyzed using Cytoscape (version 3.6.0), a bioinformatics visualization software.

### 2.4 Statistical analysis

The efficacy and safety outcomes were analyzed using Comprehensive Meta-Analysis Software (version 3.7). The effect sizes for all combined outcomes were expressed as 95% confidence intervals (CI), with upper and lower limits. Heterogeneity among the studies was assessed using the I^2^ statistic. A random-effect model was employed when I^2^ exceeded 50%, while a fixed-effect model was used when I^2^ was 50% or lower. A P value of less than 0.05 was considered statistically significant. Sensitivity analyses were conducted by individually excluding each study highly heterogeneous from the aggregated outcomes to assess their impact on the overall results. All analyses were based on the patient data from the included trials.

### 2.5 Quality assessment of the studies

Two authors independently assessed the methodological quality of the included nonrandomized controlled trials using the Methodological Index for Nonrandomized Studies (MINORS). The MINORS tool comprises 12 assessment criteria, with the first eight criteria specifically tailored for noncomparative research. Each criterion is scored on a scale from 0 to 2, where 0 indicates that the item is not reported, 1 indicates that the item needs to be more adequately reported, and 2 indicates that the item is adequately reported. The maximum total score achievable is 16 points.

## 3 Results

### 3.1 Literature search

**FIGURE 1:**
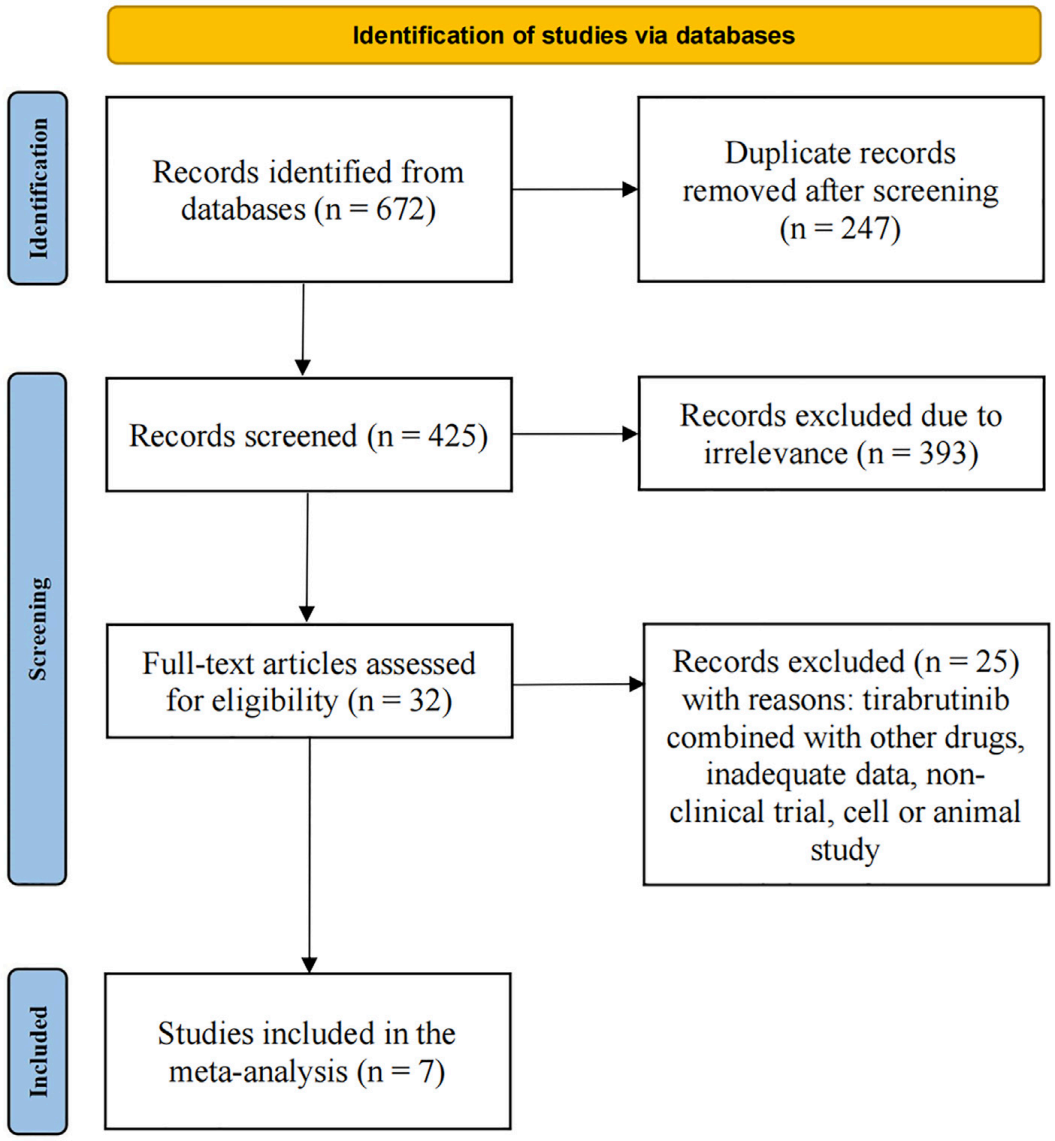
Flow chart of the literature search and selection process.

As of 20 February 2024, a total of 672 relevant articles were identified through a comprehensive search of the Embase, PubMed, Web of Science, and Cochrane Library databases. After removing duplicates, 247 articles were excluded. Applying the predefined inclusion and exclusion criteria, and conducting an initial review of titles and abstracts, an additional 393 articles were excluded. A detailed full-text review of the remaining articles led to the exclusion of a further 25 articles. Ultimately, seven articles were selected for inclusion in our meta-analysis. The screening process is illustrated in Figure 1, and the basic information of the included articles is summarized in Table 1.

**TABLE 1:** Basic information about selected articles.

Study name	Clinical trial registration number	Phase	Study design	Cancer type	Number of patients	Male/female	Median age (range)	Prior no. of therapies, median (range)
Danilov et al. (2020)	NCT02457598	Ib	Multicenter, single-arm	Relapsed/refractory chronic lymphocytic leukemia	29	17/12	70 (52–91)	1.0 (1–6)
Narita et al. (2021)	JapicCTI-173646	I/II	Multicenter, single-arm	Relapsed/refractory primary central nervous system lymphoma	44	24/20	60 (29–86)	2.0 (1–14)
Munakata et al. (2023)	JapicCTI-142682	I	Single-arm	Relapsed or refractory B-cell malignancies	17	8/9	70 (37–80)	Not mention
Sekiguchi et al. (2022)	JapicCTI-184057	II	Multicenter, single-arm	Relapsed/refractory and treatment-naïve Waldenström’s macroglobulinemia	27	22/5	71 (50–83)	Relapsed/refractory: 2.0 (1–7)
Walter et al. (2017)	NCT02457559	I	Single-arm	Relapsed/refractory chronic lymphocytic leukemia	28	23/5	67 (40–83)	3.5 (2–7)
Rule et al. (2020)	Not mention	I	Single-arm	Relapsed/refractory mantle cell lymphoma	16	12/4	64 (52–81)	3.0 (2–8)
Kawasaki et al. (2022)	jRCT2011210002	Ⅳ	Multicenter	Recurrent or refractory primary central nervous system lymphoma	140	72/68	70 (30–88)	Not mention

### 3.2 Safety

Clinical trials evaluating tirabrutinib, predominantly conducted in patients with CLL, PCNSL, MCL, and WM, have yielded valuable insights into its safety profile. Across the seven studies included in this analysis, AEs were meticulously documented and their incidence rates were accurately determined. Notably, the most frequently reported AEs of all grades were neutropenia (26.7%), diarrhea (26.0%), and rash (24.9%). Among grade ≥3 AEs, neutropenia (18.4%), lymphocytopenia (11.7%), and anemia (10.3%) were the most common.

**FIGURE 2:**
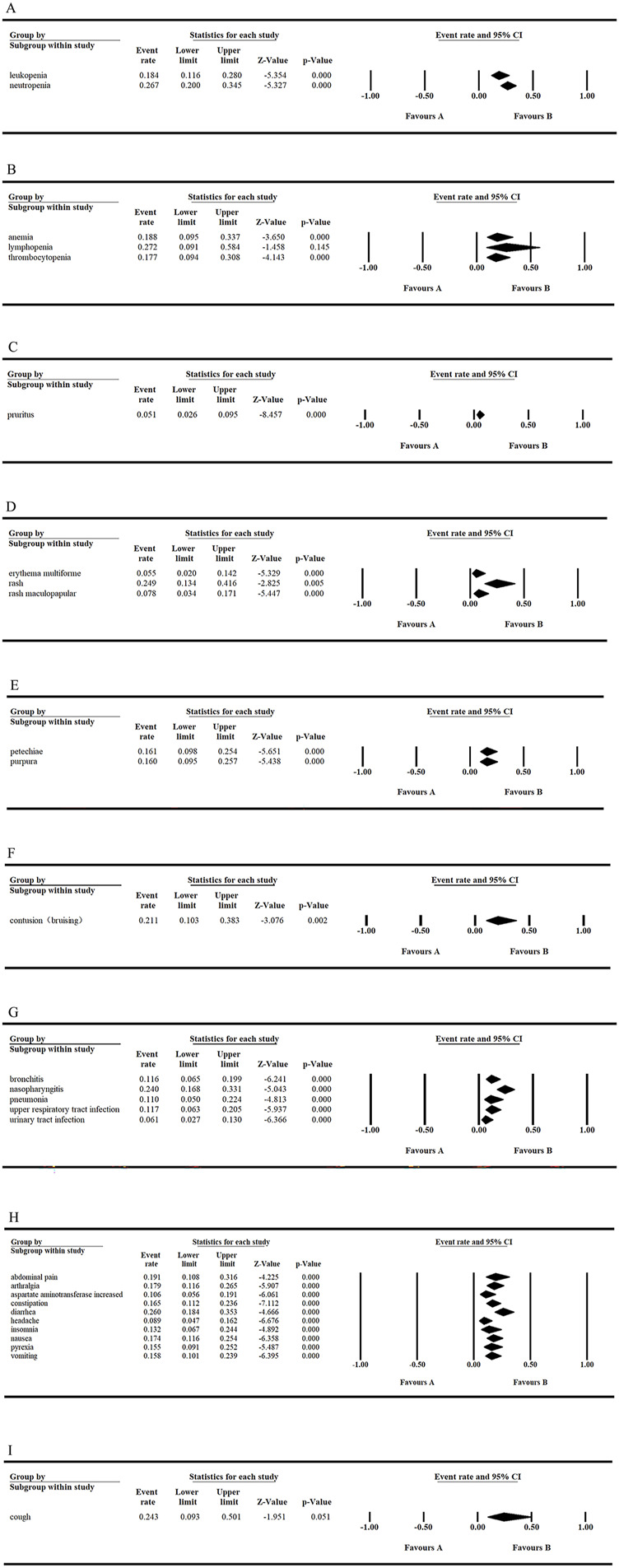
Frequency of all grades AEs in tirabrutinib monotherapy. **(A, C, E, G)** Fixed model **(B, D, F)** Random model.

**FIGURE 3:**
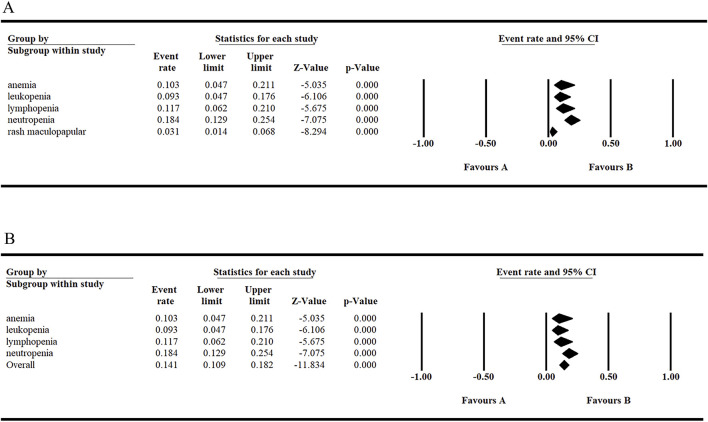
The incidence of grade ≥3 AEs and the overall probability of grade ≥3 hematologic AEs with tirabrutinib monotherapy. **(A, B)** Fixed model.

With tirabrutinib monotherapy, the three most common hematologic AEs of all grades were neutropenia (26.7%), anemia (18.8%), and leukopenia (18.4%). Among grade ≥3 hematologic AEs, neutropenia (18.4%), lymphopenia (11.7%), and anemia (10.3%) were most frequently observed. Additionally, the top three non-hematologic AEs of all grades were diarrhea (26.0%), rash (24.9%), and nasopharyngitis (24.0%). These findings are illustrated in Figures 2, 3. Among cutaneous AEs, rash had the highest incidence rate across all grades, with an overall incidence of 24.9% (95% CI: 0.134–0.416). For inflammatory AEs, nasopharyngitis was the most common across all grades, with an incidence rate of 24.0% (95% CI: 0.168–0.331). Regarding bleeding-related AEs, contusion was the most frequent, with an overall incidence rate of 21.1% (95% CI: 0.103–0.383). Cardiovascular events, which are notable side effects of covalent irreversible BTK inhibitors, are of significant clinical concern. Only two studies have reported the incidence of atrial fibrillation (AF) as a cardiovascular AE associated with tirabrutinib. For AF, Sekiguchi et al. (2022) (Sekiguchi et al., 2022) reported an incidence rate of 7%, while Danilov et al. (2020) (Danilov et al., 2020) reported a rate of 3%. It was conclude that tirabrutinib has a favorable safety profile in terms of the low incidence of AF-related AEs.

Among patients with CLL treated with tirabrutinib monotherapy, two studies reported that the most common AEs of all grades were neutropenia (35.7%, 21%), diarrhea (31.0%, 25.0%), and constipation (21.4%, 21.0%). The most common grade ≥3 AE was neutropenia (25.0%, 17.0%). In patients with PCNSL, the most common AEs of all grades were rash (31.8%, 11.4%), erythema multiforme (11.4%, 2.9%), and maculopapular rash (6.8%, 2.9%). The most common grade ≥3 AE was maculopapular rash (2.3%, 2.9%). Patients with MCL commonly experienced thrombocytopenia (44.0%), diarrhea (44.0%), and cough (44.0%) as all grades AEs, with thrombocytopenia (19.0%) being the most common grade ≥3 AE. Finally, in patients with WM, the most frequently reported AEs of all grades were rash (44.0%), neutropenia (30.0%), and nasopharyngitis (26.0%), with neutropenia (22.0%) being the most common grade ≥3 AE.

### 3.3 Efficacy

**FIGURE 4:**
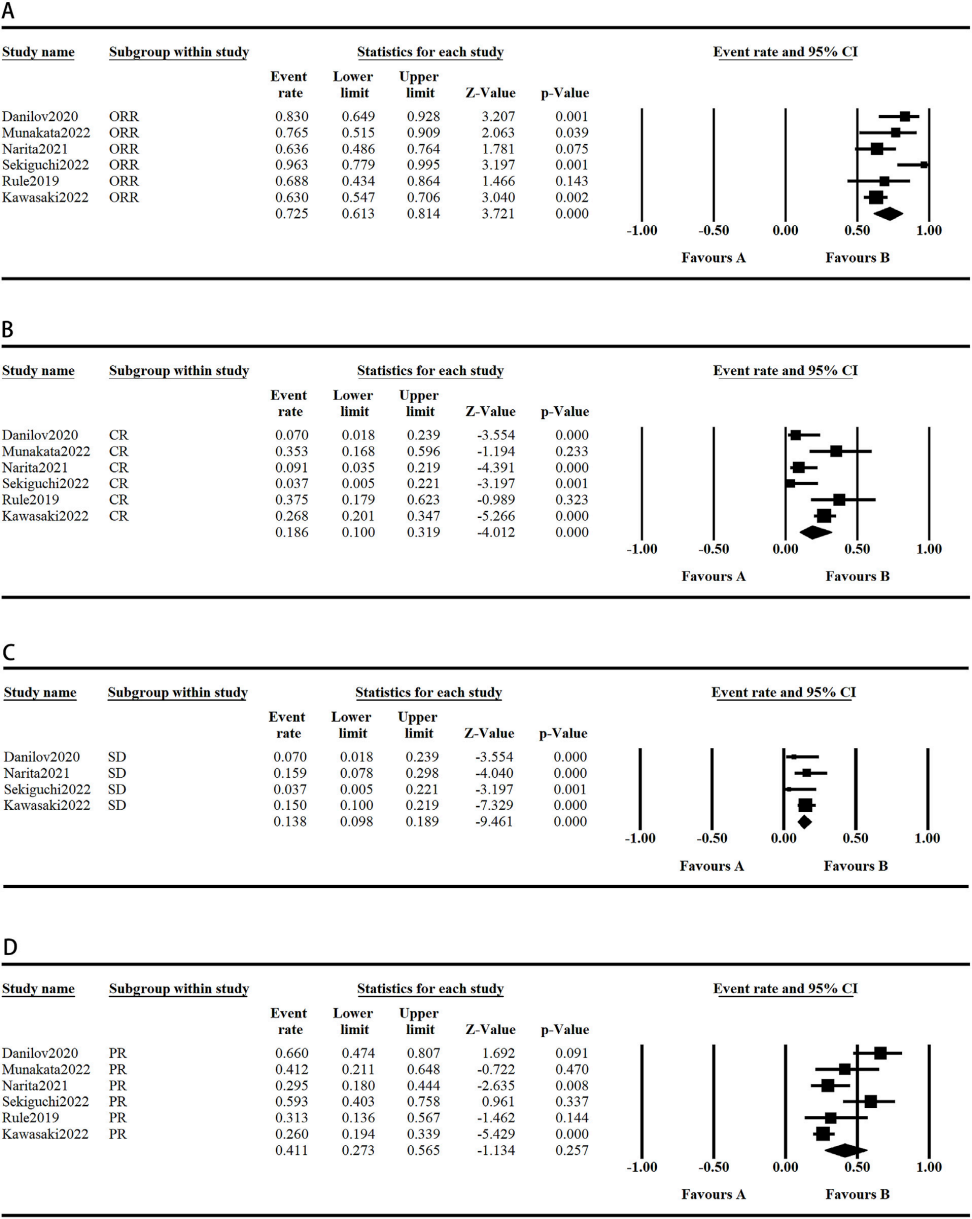
Efficacy of tirabrutinib in the treatment of B-cell lymphoma. **(A, B, D)** Random model **(C)** Fixed model.

The following survival endpoints were assessed: ORR, SD, CR, PR, and mPFS. These endpoints were evaluated based on the response criteria for patients with CLL/small lymphocytic lymphoma (SLL), as well as the response criteria for NHL established by the International Working Group. In the included studies, the longest median number of prior treatment lines was 3.5 (range: 2–7), reported by Walter et al. (2017) in patients with CLL. Conversely, the shortest median number of prior treatment lines was 1.0 (range: 1–6), reported by Danilov et al. (2020) (Danilov et al., 2020) in patients with CLL. The overall ORR across all patients was 72.5%. Notably, Sekiguchi et al. (2022) reported the highest ORR of 96.3% with tirabrutinib monotherapy in patients with WM. The overall CR rate for all patients was 18.6%, with Rule et al. (2020) achieving a CR of 37.5% in patients with MCL treated with tirabrutinib monotherapy. The overall SD rate for all patients was 13.8%, and Narita et al. (2021) reported a SD rate of 15.9% in patients with relapsed or refractory PCNSL treated with tirabrutinib monotherapy. The overall PR rate for all patients was 41.1%, although this result should be interpreted with caution due to its complexity. In a study of CLL, Danilov et al. (2020) reported the highest PR rate of 66% with tirabrutinib monotherapy (Figure 4). Lastly, Walter et al. (2017) reported the longest mPFS of 38.5 months in patients with CLL treated with tirabrutinib.

### 3.4 Sensitivity analysis

**FIGURE 5:**
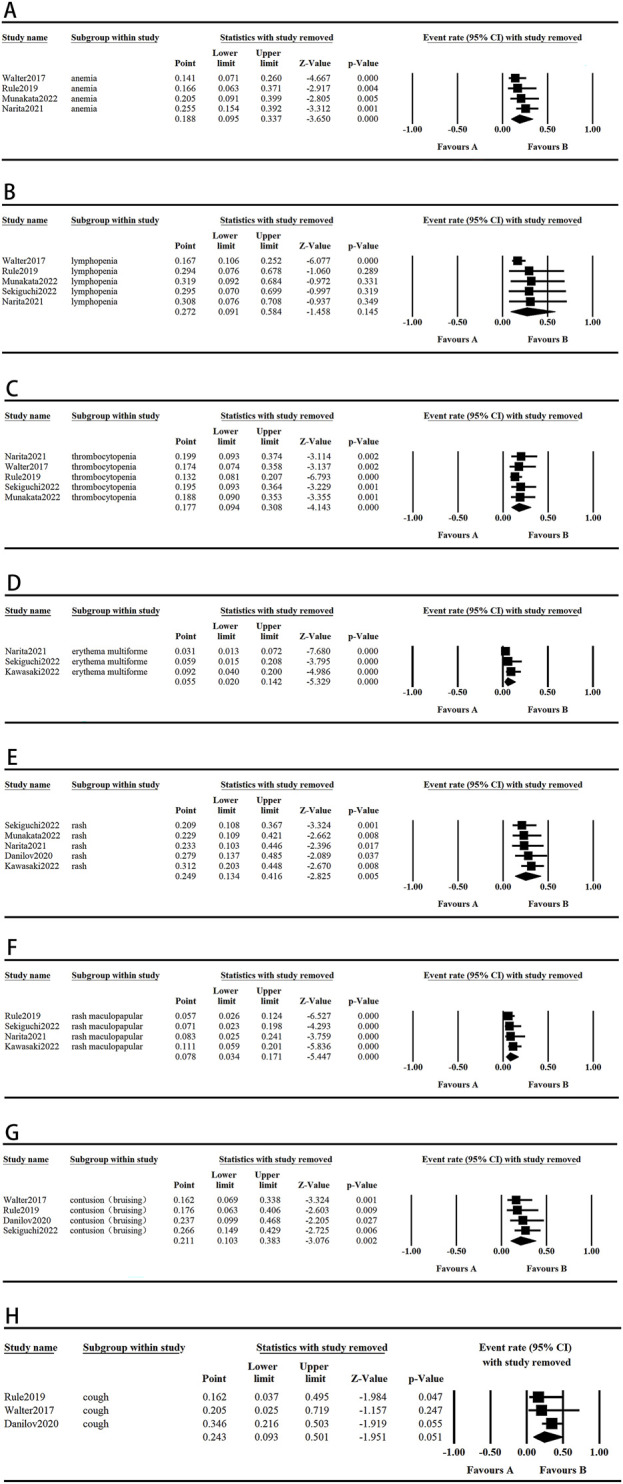
The graph of the safety sensitivity analysis. (**A-H**) Random model.

**FIGURE 6:**
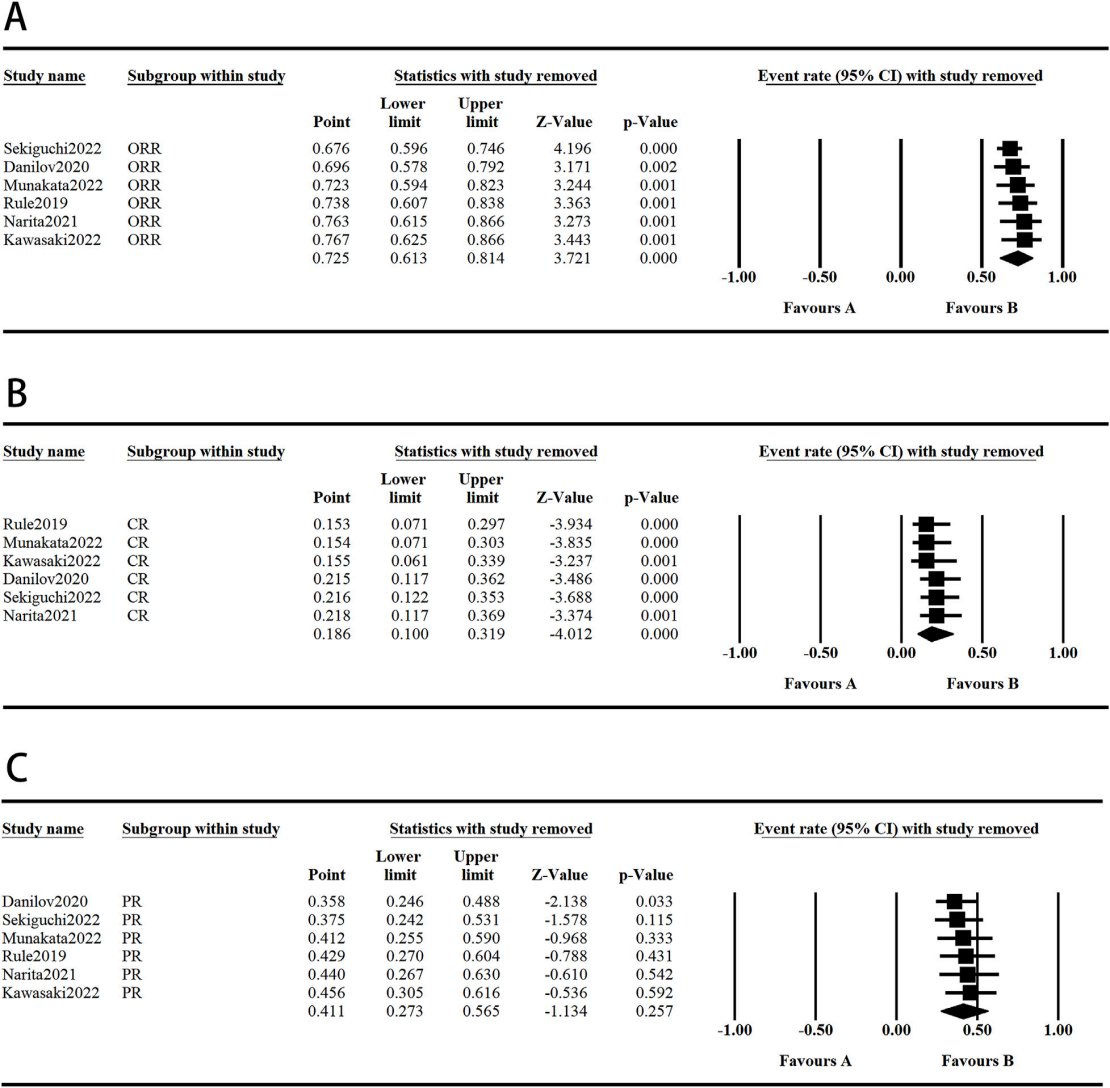
The graph of the efficacy sensitivity analysis. (**A-C**) Random model.

The sensitivity analysis was conducted by excluding individual studies from the aggregated outcomes, which exhibited high levels of heterogeneity, to assess their impact on the overall results. The results for lymphopenia, cough, and PR rate were not robust due to notable variations in sample sizes across studies, as well as the presence of individual heterogeneity within each study. In contrast, the pooled analysis of other outcomes did not show significant variations, suggesting that our comprehensive results maintain a high degree of reliability and consistency. These results are shown in Figures 5, 6.

### 3.5 Pharmacokinetics

Pharmacokinetic parameters included T_max_, T_1/2_, and C_max_. When patients received tirabrutinib monotherapy at a daily dose of 80 mg, Walter et al. (2016) reported a T_max_ of 1–2 h, a T_1/2_ of 4–7 h, and a C_max_ of 250–400 ng/mL for CLL. Munakata et al. (2019) reported that for B-cell malignancies, the T_max_ was 1–6 h, the T_1/2_ was 3.5–6 h, and the C_max_ was 550–700 ng/mL. Danilov et al. (2020) reported that for CLL, the T_max_ was 1–2 h, the T_1/2_ was 4–7 h, and the C_max_ was 200–300 ng/mL (Table 2).

**TABLE 2:** Pharmacokinetics of tirabrutinib in various studies.

Study name	T_max_	T_1/2_	C_max_
Walter et al. (2016)	1–2 h	4–7 h	250–400 ng/mL
Munakata et al. (2019)	1–6 h	3.5–6 h	550–700 ng/mL
Danilov et al. (2020)	1–2 h	4–7 h	200–300 ng/mL

### 3.6 Target genes

**FIGURE 7:**
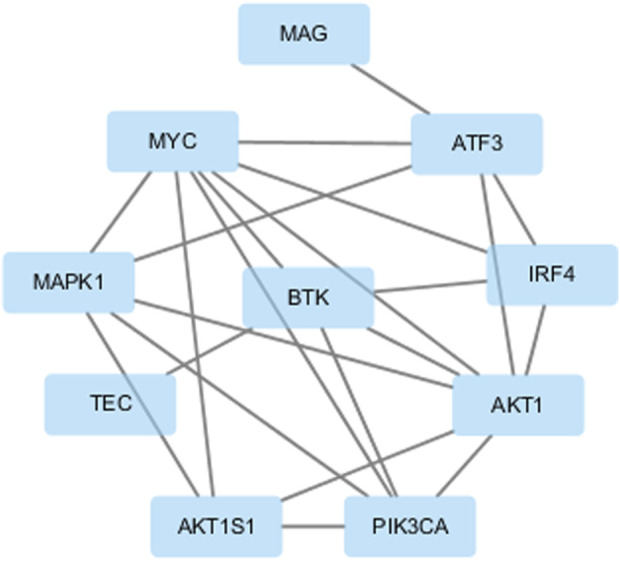
The target genes of tirabrutinib.

The target genes of tirabrutinib identified in published studies primarily encompass the BTK signaling pathway, including BTK, TEC, as well as the PI3K/AKT/mTOR signaling pathway, comprising PI3K, AKT, and mTORC1. Notably, BTK, AKT, and PI3K are well-established targets implicated in B-cell lymphoma and leukemia, while MYC is also a key oncogene associated with these diseases. KEGG pathway enrichment analysis indicates that these genes are involved in multiple pathways. Among these, pathways related to hematological malignancies include acute myeloid leukemia, chronic myeloid leukemia, and Epstein-Barr virus infection, which is known to be associated with certain lymphomas. GO enrichment analysis indicated that tirabrutinib promotes cancer cell death by regulating cell proliferation, differentiation, and multiple signaling pathways (Supplementary Figures S1, S2). The PPI network is shown in Figure 7.

### 3.7 Qualitative assessment of the study

A qualitative assessment was performed using the MINORS criteria to evaluate the quality of the included non-randomized controlled trials. The MINORS scores of the seven studies ranged from 8 to 12 points, indicating that the overall quality of these studies is satisfactory. A more detailed overview is provided in Table 3.

**TABLE 3:** The MINORS scores of seven single-arm trials.

Study	1	2	3	4	5	6	7	8	Total
Danilov et al. (2020)	2	2	1	2	0	1	2	0	10
Narita et al. (2021)	2	2	1	2	0	1	2	0	10
Caldeira et al. (2019)	2	2	1	2	0	2	2	0	11
Sekiguchi et al. (2022)	2	2	1	2	0	2	2	0	11
Walter et al. (2017)	2	2	1	2	0	2	2	0	11
Rule et al. (2020)	2	2	2	2	0	2	2	0	12
Gupta et al. (2025)	2	2	2	2	0	0	0	0	8

## 4 Discussion

The BCR signaling pathway is crucial in the development of B-cell-derived hematologic malignancies, including CLL, diffuse large B-cell lymphoma, MCL, and other NHL. BTK, a kinase of the TEC family, plays multiple roles in the BCR signaling pathway and is a critical therapeutic target for treating B-cell malignancies. Initially, BTK was identified as a potential contributor to the pathogenesis of X-linked agammaglobulinemia, an inherited immunodeficiency disorder. Mutations in the BTK gene cause impaired maturation of pre-B cells in the bone marrow, preventing their development into mature B cells that migrate to peripheral tissues (Alu et al., 2022; Wu et al., 2016). The first BTK inhibitor, ibrutinib, was approved by the U.S. Food and Drug Administration (FDA) in 2013 for treating relapsed or refractory MCL. This approval marked a significant milestone in the treatment of hematologic cancers by introducing chemotherapy-free treatment options. Ibrutinib is now also used to treat CLL/SLL, WM, and marginal zone lymphoma, becoming a cornerstone in the management of B-cell lymphomas (Muñoz et al., 2023). However, ibrutinib’s off-target activity and acquired resistance to inhibition of other kinases have driven the development of second-generation BTK inhibitors with improved selectivity (Liclican et al., 2020; Lin and Andreotti, 2023; Bond and Woyach, 2019).

Tirabrutinib is a recently developed, highly selective, oral second-generation BTK inhibitor that irreversibly binds to BTK C481. It exhibits an IC50 of 6.8 nM for BTK and 48 nM for TEC, demonstrating significantly higher selectivity compared to other kinases such as ITK (IC50 > 20,000 nM) (Makita et al., 2020). Tirabrutinib has shown clinical efficacy against multiple relapsed or refractory B-cell malignancies (Liclican et al., 2020), inducing durable responses without significant toxicity, potentially offering advantages in reducing toxicity due to its more selective kinase inhibition (Walter et al., 2016). It has been approved for the treatment of relapsed or refractory PCNSL (Schaff et al., 2024; Okita et al., 2021) and WM (Castillo et al., 2020)/lymphoplasmacytic lymphoma (Kozaki et al., 2023). Although tirabrutinib has been administered to patients with B-cell lymphomas, its efficacy and safety require further scientific validation. Therefore, it is crucial to accurately identify patients who would benefit from BTK inhibitor therapy and to implement effective screening and management strategies for AEs throughout treatment. To this end, a meta-analysis was conducted, pooling data from seven clinical trials involving a total of 301 patients with CLL, PCNSL, MCL, and WM, to evaluate the safety and efficacy of tirabrutinib in various B-cell lymphomas. The overall ORR, CR, SD, and PR rates were 72.5%, 18.6%, 13.8%, and 41.1%, respectively. Among these, patients with WM had the highest ORR at 96.3%. Analysis of AEs revealed that neutropenia was the most common AE of all grades and grade ≥3. Diarrhea was the most common non-hematologic AE across all grades. CLL patients exhibited the highest incidence of neutropenia, while PCNSL and WM patients were most susceptible to skin-related AEs. MCL patients had the highest risk of thrombocytopenia. Long-term follow-up data from the majority of included studies demonstrated that tirabrutinib maintained its efficacy with good tolerability and no new safety or toxicity concerns. However, some studies suggested that extended follow-up is necessary to fully validate the long-term efficacy of tirabrutinib. Our analysis indicates that tirabrutinib is an effective treatment for B-cell lymphomas, providing significant clinical benefits. Nonetheless, clinicians should remain vigilant in monitoring and managing side effects specific to each type of disease.

TP53 mutations and del (17p) are among the most common adverse prognostic factors in CLL/SLL, closely associated with accelerated disease progression, increased treatment resistance, and shorter PFS. For instance, in the study Danilov et al. (2020), BTK inhibitors significantly improved PFS compared to conventional chemoimmunotherapy regimens (bendamustine plus rituximab; fludarabine, cyclophosphamide, and rituximab; and chlorambucil), particularly in patients with TP53 mutations and del (17p). Among 12 high-risk patients 10 achieved CR or PR, while 2 did not respond. This highlights the significant therapeutic advantage of BTK inhibitors in treating high-risk patients who often fail to respond to traditional therapies. In the study Walter et al. (2017), 13 of 25 patients (52%) exhibited TP53 mutations, and these high-risk patients responded well to BTK inhibitors. Unmutated immunoglobulin heavy-chain variable region (IGHV) gene status is also closely linked to disease aggressiveness and poor prognosis. According to Danilov et al. (2020), 21 of 25 patients (84%) had unmutated IGHV gene, and these patients demonstrated favorable responses to BTK inhibitors.

BTK inhibitors, including ibrutinib, acalabrutinib, zanubrutinib, tirabrutinib, and orelabrutinib, have been approved for clinical use and are all covalent, irreversible BTK inhibitors. On 27 January 2023, the FDA approved the non-covalent BTK inhibitor pirtobrutinib for treating relapsed or refractory MCL, heralding the advent of non-covalent BTK inhibitors (De, 2023). The first-generation BTK inhibitor, ibrutinib, is the pioneering covalent, irreversible BTK inhibitor approved for treating various B-cell malignancies, including CLL, MCL, and WM. While ibrutinib has demonstrated significant clinical efficacy, its covalent binding to BTK can lead to off-target effects, resulting in AEs such as atrial fibrillation, hypertension, and bleeding (Karimi et al., 2025). In contrast, tirabrutinib is a second-generation BTK inhibitor that offers several pharmacological advantages: it exhibits higher selectivity for BTK compared to first-generation inhibitors like ibrutinib, thereby reducing off-target effects and associated AEs. Additionally, tirabrutinib has a longer half-life, which allows for more stable drug concentrations and potentially lower dosing frequencies. This pharmacokinetic profile helps maintain therapeutic efficacy while reducing the risk of AEs related to fluctuations in drug concentrations (Tavakoli et al., 2024). Compared to other second-generation BTK inhibitors such as acalabrutinib and zanubrutinib, tirabrutinib has demonstrated comparable efficacy in treating relapsed or refractory B-cell malignancies while maintaining a favorable safety profile (Mushtaq et al., 2024). One study reported ORR of 78% for zanubrutinib and 74% for ibrutinib in treating CLL (Liu et al., 2023), suggesting that tirabrutinib may offer superior efficacy compared to these agents. Two additional studies showed ORRs of 82% (Park et al., 2024) and 87% (Yin et al., 2024) for acalabrutinib in CLL treatment, which aligns with our findings. Neutropenia and infections were common AEs in these studies, consistent with our conclusions. Overall, tirabrutinib’s high selectivity, improved safety and efficacy, favorable pharmacokinetics, and reduced off-target effects position it as a promising therapeutic option for patients with B-cell malignancies (Gupta et al., 2025).

The introduction of BTK inhibitors has revolutionized the treatment landscape of B-cell lymphomas by disrupting the pathological mechanisms in malignant B cells, leading to improved drug response and reduced toxicity (Wang et al., 2021). Second-generation BTK inhibitors are designed to be more selective, but they can still lead to notable side effects. For example, acalabrutinib has been associated with headaches and cough, while zanubrutinib and tirabrutinib have been linked to neutropenia. Each drug has a distinct toxicity profile that requires tailored management strategies (Makita et al., 2020). Therefore, physicians must select the most appropriate inhibitor for each patient, considering the various safety considerations.

Newer drugs targeting the BCR pathway have demonstrated a more favorable toxicity profile compared to traditional chemoimmunotherapy. However, there is still significant room for improvement in terms of response depth and duration. The combination of targeted agents holds promise for shorter treatment durations while enhancing response rates (Kutsch et al., 2022). For instance, the combination of idelalisib and tirabrutinib has shown synergistic inhibitory effects on the growth of diffuse large B-cell lymphoma and MCL cell line subsets. Specifically, this combination exhibited a more substantial suppression of cell proliferation in A20 mutant TMD8 cells compared to idelalisib alone. This suggests that combination therapy can bypass specific BTK pathway resistance mechanisms. As a novel inhibitor, tirabrutinib offers new targeted treatment options for B-cell lymphomas. Further research into combination therapies with other drugs and novel small molecule inhibitors targeting different pathways will likely lead to less toxic and more targeted treatment options for B-cell malignancies, thereby improving clinical efficacy (Wu et al., 2016; Akinleye et al., 2013).

It is important to acknowledge that our analysis is subject to certain limitations. The studies included in the analysis were not designed according to the principles of randomized controlled trials. The scope of our analysis was limited to data from completed or fully published studies. Additionally, the impact of drug dosage on these outcomes was not evaluated. Notably, patients in the study received varying doses of the drug, and some AEs may be dose-dependent. Given the limited number of clinical trials investigating tirabrutinib monotherapy in B-cell lymphoma, further large-scale, randomized, high-quality, controlled trials are essential to validate the findings of this study.

## Data Availability

The original contributions presented in the study are included in the article/Supplementary Material, further inquiries can be directed to the corresponding author.

## Abbreviations

BTK, Bruton’s tyrosine kinase; CLL, chronic lymphocytic leukemia; PCNSL, primary central nervous system lymphoma; MCL, mantle cell lymphoma; WM, Waldenstrom’s Macroglobulinemia; mPFS, median progression-free survival; ORR, overall response rate; CR, complete remission; SD, stable disease; PR, partial response; AE, adverse event.
